# Experience with genomic sequencing in pediatric patients with congenital cardiac defects in a large community hospital

**DOI:** 10.1002/mgg3.357

**Published:** 2018-01-25

**Authors:** Natalie S. Hauser, Benjamin D. Solomon, Thierry Vilboux, Alina Khromykh, Rajiv Baveja, Dale L. Bodian

**Affiliations:** ^1^ Inova Translational Medicine Institute Falls Church VA USA; ^2^ Inova Children's Hospital Inova Health System Falls Church VA USA; ^3^ Present address: GeneDx Gaithersburg MD USA

**Keywords:** congenital heart disease, genetic, whole genome sequencing

## Abstract

**Background:**

Congenital cardiac defects, whether isolated or as part of a larger syndrome, are the most common type of human birth defect occurring on average in about 1% of live births depending on the malformation. As there is an expanding understanding of the underlying molecular mechanisms by which a cardiac defect may occur, there is a need to assess the current rates of diagnosis of cardiac defects by molecular sequencing in a clinical setting.

**Methods and Results:**

In this report, we evaluated 34 neonatal and pediatric patients born with a cardiac defect and their parents using exomized preexisting whole genome sequencing (WGS) data to model clinically available exon‐based tests. Overall, we identified candidate variants in previously reported cardiac‐related genes in 35% (12/34) of the probands. These include clearly pathogenic variants in two of 34 patients (6%) and variants of uncertain significance in relevant genes in 10 patients (26%), of these latter 10, 2 segregated with clinically apparent findings in the family trios.

**Conclusions:**

These findings suggest that with current knowledge of the proteins underlying CHD, genomic sequencing can identify the underlying genetic etiology in certain patients; however, this technology currently does not have a high enough yield to be of routine clinical use in the screening of pediatric congenital cardiac defects.

## 1 INTRODUCTION

Congenital cardiac defects, whether they arise as isolated defects or occur in association with an underlying syndrome, are the most common birth defects in newborns. The incidence of severe congenital cardiac defects is estimated to be as low as 6/1,000 live births but ranges up to 75/1,000 if minor or asymptomatic defects are considered (Hoffman, 1995; Hoffman & Kaplan, 2002). The underlying molecular cause of congenital cardiac defects is not well understood although genetic, epigenetic, and environmental factors have been implicated. Over the last two decades, intensive efforts have been employed to find new solitary or aggregate underlying genetic etiologies to explain congenital cardiac defects. Recently, using genetic technologies including genomic sequencing and microarrays, the pursuit to clarify genetic etiologies for aberrant cardiac development has intensified. Due to these ongoing efforts, there is an evolving understanding of the underlying means by which a cardiac defect may occur (Iascone et al., 2012; Jia et al., 2015; Jin et al., 2017; Zaidi & Brueckner, 2017).

Previous studies aimed at identifying causative mutations in patients with congenital heart defects have focused on characterizing variants in limited numbers of genes. Iascone et al. (Iascone et al., 2012) using a genomic approach combining candidate gene sequencing of five genes and genome‐wide array comparative genomic hybridization, evaluated 53 patients with isolated hypoplastic left heart syndrome and found 20 patients (37%) who had either multiple copy number variants (CNVs) or one point mutation and at least one CNV. Jia et al. (Jia et al., 2015) investigated the coding regions of 57 genes associated with nonsyndromic autosomal dominant congenital heart defects (CHD) by targeted sequencing in 36 patients from 13 nonsyndromic CHD families. They identified six potential disease‐causing variants in three genes (*MYH6, NOTCH1*, and *TBX5*) that may explain the defects in six families (46%). Since these approaches have not identified causative variants for most patients, attention is turning toward looking at a wider set of genes as a more comprehensive method for exploring genetic variation as a cause of congenital cardiac defects.

In this report, we describe a cohort of 34 pediatric patients with congenital cardiac defects either in isolation or associated with other noncardiac abnormalities. Our cohort is representative of the patients a clinician may see in any community hospital, unfiltered by selection or family history. All of the patients as well as both of their parents underwent research‐based whole genome sequencing (WGS) from which exome like regions were analyzed to simulate trio‐based clinical testing. The results illustrate successful diagnoses as well as issues encountered in interpreting the results from NGS, which can be considered by practitioners when applying DNA sequencing technologies for this class of disorders.

## 2 MATERIALS AND METHODS

### 2.1 Participants and clinical phenotypes

The subjects for this analysis participated in one of two ongoing IRB‐approved research studies conducted by the Inova Translational Medicine Institute (ITMI) located at the Inova Fairfax Medical Center. The studies “The Incidence and Burden of Congenital Anomalies, Genetic Disorders and Genetic Suspicion in Neonatal and Pediatric Patients” and “Genomic Correlations to Childhood Health Outcomes: The First 1,000 Days of Life” were both approved by the Western Institutional Review Board (WIRB# 20121680 and #20120204) and the Inova Institutional Review Board (IRB#15‐18196 and #15‐1804), with full informed consent obtained for all participants (the parents provided consent for the probands). In the first research protocol, we recruited neonatal and pediatric patients with congenital birth defects of all types, in whom, at the time of enrollment, an underlying etiology for the suspected genetic disorder had not been found using conventional genetic testing methods. Participants were recruited prenatally (1%), or from the neonatal intensive care unit (NICU) (~66%), pediatric intensive care unit (PICU) (~31%), and the general inpatient pediatric wards (~3%) of Inova Children's Hospital, Falls Church, VA, USA. Inclusion criteria included the availability of samples from both biological parents for WGS concurrent with the proband. From this larger cohort, the cardiac subcohort was composed of neonates with cardiac defects either in isolation or as part of a suspected but etiologically unidentified syndrome. The second research protocol, “Genomic Correlations to Childhood Health Outcomes: The First 1,000 Days of Life” enrolled healthy pregnant females and their partners recruited from local obstetric offices prior to delivery. Participants were unselected for genetic disorders. All infants with cardiac defects without known etiology from that longitudinal cohort study were included in our cardiac subcohort. Characterization of cardiac defects included transthoracic echocardiography in all probands in the subcohort. Family members were phenotyped using self‐reported questionnaires as well as information derived through clinical genetic consults. Biological parentage was confirmed using the genomic sequences, and ancestry was calculated from the genomic data as described (Bodian et al., 2014).

### 2.2 Whole genome sequencing and analysis

Genomic DNA was extracted from peripheral white blood cells from all individuals and WGS was performed as described (Khromykh et al., 2015). Filtering for candidate pathogenic variants was conducted with an internally developed analysis pipeline and two commercial pipelines, Ingenuity Variant Analysis (QIAGEN Inc., Valencia, CA, USA), and Personalis Research Analysis Service (Personalis, Inc., Menlo Park, CA, USA), both applied by the vendors. For the internal pipeline, small variants were annotated with ANNOVAR version 2014‐07‐14 (Wang, Li, & Hakonarson, 2010) using RefSeq transcripts, (O'Leary et al., 2016) frequency data from the 1000 Genomes Project, (Auton et al., 2015) and ExAC (Lek, Karczewski, Minikel, Samocha, & Banks, 2016) as provided in the ANNOVAR database popfreq_all_20150413, and from internal databases (Bodian et al., 2016). Pathogenicity data were extracted from the ClinVar 08/2016 xml file (Granados‐Riveron et al., 2012) and HGMD Professional version 2015.3 (Stenson et al., 2014). Conservation of variants across species was obtained from the multiz (Blanchette et al., 2004) alignment of 100 vertebrates provided by the UCSC Genome Browser (Kent et al., 2002). Candidate variants were required to lie in genes previously associated with cardiac conditions, using a predefined gene list (Table S1) created in part by genes noted in the following publications (Lalani & Belmont, 2014; Topf et al., 2014), complemented by literature searches of genes with candidate variants that pass the filtering criteria. The variants were required to have low frequency (<1%) in control populations, and to meet published sequencing quality criteria (Bodian et al., 2017). Potentially damaging variants in known cardiac genes were defined as rare variants (MAF <0.001 in gnomAD) (Lek et al., 2016) that were either predicted to be deleterious by MetaSVM (Kim, Jhong, Lee, & Koo, 2017), or annotated as splice site, frameshift, nonframeshift, or stopgain (Table S2). Potentially positive results identified through any pipeline were individually evaluated by our group of clinical and molecular geneticists and bioinformaticists for likelihood of pathogenicity following the ACMG guidelines (Richards et al., 2015).

For the internal pipeline, CNVs were called using the Reference Coverage Profiles method (Glusman et al., 2015) then filtered for those considered candidates for causality. Candidate CNVs were required to have frequency <0.05 in a predominantly healthy control population, (Bodian et al., 2016) and to overlap the coordinates of at least one exon of a gene implicated in cardiac disease.

## 3 RESULTS

### 3.1 Clinical description

Thirty‐four patients and their parents were evaluated in this study (Table 1). The proband group consists of 17 males and 17 females, all with a primary cardiac defect. Sixteen (47%) of the patients had at least one additional extracardiac finding, and nine (26%) had two additional findings; these latter nine subjects (2, 3, 7, 8, 13, 18, 24, 28, 34) were considered syndromic patients. The two types of cardiac defects seen most frequently among the entire cohort were tetralogy of Fallot (seven patients) and transposition of the great arteries (nine patients, three associated with double outlet right ventricle, one associated with dextrocardia). Hypoplastic left heart defect was the next most frequent with four patients affected. The two types of cardiac defects seen most frequently in combination with two or more extracardiac physical finding were also transposition of the great arteries and tetralogy of Fallot. Transposition of the great arteries was seen with various combinations of the following physical findings in four patients (3, 13, 30, and 34): dysmorphic facial features, hypospadias, intrauterine growth restriction, hydronephrosis, preauricular ear tag, and heterotaxy. Tetralogy of Fallot was seen along with various combinations of the following findings in three patients (8, 18, and 24): choanal atresia, hypoplasia of the semicircular canals, vertebral body anomalies, learning disabilities, jejunal atresia, and preauricular skin tag. The cohort was ancestrally diverse, with 13 Caucasian probands (38%), seven with more than one ancestral category (21%), five Hispanic (15%), five African American (15%), two Asian (6%), and one Middle Eastern (3%). All patients except for two (11 and 34) were index patients with no reported family histories of cardiac defects. Seven pregnancies (21%) were complicated by diabetes mellitus, three (9%) with gestational diabetes (5, 17, 24), and four (11%) with diabetes preceding pregnancy (3, 6, 26, 34).

**Table 1:** Patient features and genomic findings

Patient	Gender	Cardiac defect	Other features	Clinical testing	Prioritized candidate variants	ACMG classification	Inheritance	Family members affected	Variant confirmation
1	Male	Atrial flutter, medium ASD, tricuspid regurgitation, PDA, right ventricular hypertension.	Hypoglycemia, large for gestational age	Normal methylation for Beckwith‐Wiedemann^b^, normal microarray^f^	*MYH6* c.899‐2A>G NM_002471.3	VUS	Maternal	None	Sanger
2	Female	Ebstein anomaly of the tricuspid valve	Hydronephrosis, Wolff Parkinson White	Microarray^a^ with Xp22.11 deletion (23,008,799–23,134,236)	*CITED2* c.574A>G p.Ser192Gly NM_006079.4	VUS	Maternal	None	Sanger
3	Male	Transposition of the great arteries	Clinodactyly of 5th finger, upslanting palpebral fissures, thickened nasolabial folds, simplified palmar creases. Infant of a diabetic mother.	Normal karyotype and microarray^a^	*NIPBL* c.5863‐180_5971 + 111del NM_133433.3	N/A	De novo	None	Failed validation
4	Female	Hypoplastic Left Heart	Postaxial foot polydactyly	Normal microarray^a^	N/A	N/A	N/A	None	N/A
5	Male	Hypoplastic left heart	None	Normal microarray^a^	*FOXL1* c.926A>G p.Asn309Ser NM_005250.2	VUS	Paternal	None	Sanger
6	Male	Coarctation of the aorta, ventricular septal defect	Multicystic kidney disease on left, hypoplastic kidney on right, infant of a diabetic mother, large for gestational age.	Normal karyotype and microarray^a^	*GATA4* c.1037C>T p.Ala346Val NM_002052.3	VUS	Maternal	None	Sanger
7	Male	Pulmonary stenosis	Broad forehead, deeply set eye	Noonan panel^c^ (11 genes) negative	N/A	N/A	N/A	None	N/A
8	Male	Tetralogy of Fallot	Choanal atresia, hypoplasia of the semicircular canal	Normal microarray^a^, CHD7^d^ c.5051‐1G>A;	*CHD7* c.5051‐1G>A NM_017780.3	Likely pathogenic	De novo	None	N/A
9	Female	Double outlet right ventricle, transposition of the great arteries	Hydronephrosis	Normal microarray^a^	*NODAL* c.397C>T p.Gln133a NM_018055.4; *CITED2* c.A245G p.His82Arg NM_006079.4	VUS/VUS	Maternal/Paternal	None	Sanger
10	Male	Transposition of the great arteries	None	Microarray^a^ with heterozygous 1q25.2 duplication (180,143,425–180,257,907)	N/A	N/A	N/A	None	N/A
11	Female	Ebstein anomaly of the tricuspid valve	None	Normal microarray^a^	*MYH7* c.722C>T p.Ser241Phe NM_000257.2	VUS	Paternal	Father with mild Ebstein	Sanger
12	Male	Truncus arteriosus	None	Microarray^a^ with 4q24 202 kb deletion (102,831,284–103,033,486)	N/A	N/A	N/A	None	N/A
13	Male	Transposition of the great arteries	Hypospadias, intrauterine growth restriction	Microarray^a^ with VOUS: arr[hg19] Xp22.33 or Yp11.32(2,128,347–2,366,017 or 2,078,347–2,316,017)	N/A	N/A	N/A	None	N/A
14	Male	Tetralogy of Fallot	None	Normal microarray^a^	N/A	N/A	N/A	None	N/A
15	Female	Shone Complex	None	Normal microarray^a^	N/A	N/A	N/A	None	N/A
16	Female	Double aortic arch, vascular ring,	Subglottic stenosis from vascular ring	Not done	*GDF1* c.G485A p.Gly162Asp NM_001492.5	VUS	Paternal	None	Sanger
17	Female	Hypoplastic right heart	None	Normal microarray^a^	N/A	N/A	N/A	None	N/A
18	Female	Tetralogy of Fallot	VATER, vertebral body anomalies, learning disabilities	Not done	N/A	N/A	N/A	None	N/A
19	Male	Tetralogy of Fallot	None	Not done	N/A	N/A	N/A	None	N/A
20	Female	Coronary artery fistula to the right atrium	None	Not done	N/A	N/A	N/A	None	N/A
21	Male	Shone Complex	None	Microarray^a^ with 4q31.21q31.22 duplication (146,444,570–146,930,543)	N/A	N/A	N/A	None	N/A
22	Female	Double outlet right ventricle, transposition of the great arteries	None	Normal FISH for deletion 22q11.2, and microarray^a^	N/A	N/A	N/A	None	N/A
23	Male	Pulmonary atresia, ventricular septal defect	None	Not done	N/A	N/A	N/A	None	N/A
24	Male	Tetralogy of Fallot	Jejunal atresia, preauricular skin tag	Normal microarray^a^; *JAG1* e c.551G>A,p.R184H	*JAG1* c.551G>A p.Arg184His NM_000241.2	Pathogenic	De novo	None	N/A
25	Female	Double outlet right ventricle, transposition of the great arteries	Unilateral cleft lip and palate	Normal karyotype, FISH for 22q11.2, and microarray^a^	N/A	N/A	N/A	None	N/A
26	Female	Total anomalous pulmonary venous return	None	Normal microarray^a^	N/A	N/A	N/A	None	N/A
27	Female	Transposition of the great arteries	None	Normal microarray^a^	N/A	N/A	N/A	None	N/A
28	Male	Coarctation of the aorta	Hypospadias, intrauterine growth restriction	Normal microarray^a^	N/A	N/A	N/A	None	N/A
29	Female	Hypoplastic left heart	High palate	Not done	N/A	N/A	N/A	None	N/A
30	Female	Transposition of the great arteries	Hydronephrosis	Normal microarray^a^	N/A	N/A	N/A	None	N/A
31	Female	Tetralogy of Fallot	None	Normal microarray^a^	N/A	N/A	N/A	None	N/A
32	Male	Hypoplastic left heart variant, hypoplastic mitral and aortic valve, trivial aortic stenosis	None	Not done	*NKX2‐5* c.C494T p.Ala165Val NM_004387.3	VUS	Paternal	None	Sanger
33	Female	Tetralogy of Fallot	None	Not done	N/A	N/A	N/A	None	N/A
34	Male	Transposition of the great arteries, dextrocardia	Preauricular skin tag, heterotaxy	Normal microarray^a^	*GATA6* c.C15G p.Asp5Glu NM_005257.4	VUS	Paternal	Father with bicuspid aortic valve	Sanger

VUS, variant of unknown significance.

a Quest Diagnostics.

b Mayo Medical Laboratories, Rochester, MN.

c GeneDx, Gaithersburg, MD.

d Ambry Genetics, Aliso Viejo, CA.

e Baylor Medical Genetics Laboratory, Houston, TX.

f Progenity, Ann Arbor, MI.

Clinical genetic testing was performed prior to enrollment in the research study for the majority (25/34, 73%) of patients (Table 1). The most frequently ordered test was commercial microarray (ClariSure Oligo‐SNP, Quest Diagnostics Nichols Institute, Chantilly, VA); single‐gene testing was performed in addition to the microarrays for two patients, FISH or methylation testing for a particular syndrome was performed in three patients, and in one patient, a gene sequencing panel was performed. Of the 24 patients who had clinical microarrays performed, there were no positive findings, and five of these patients were found to have variants of unknown significance (VUS's) (Table 1). The variants were evaluated first by the commercial genetic testing laboratory and then by secondary review by the authors. In all five patients, the VUS's found on microarray were determined to be unlikely or uncertain to be contributory to the development of the cardiac defects and other clinical features. This determination was made if there was no convincing evidence that the genes included in the copy number variants were related to cardiac development, and/or the genes involved are not currently known to be disease‐associated. Parental samples were not analyzed to assess the inheritance of these CNVs deemed to be of low clinical suspicion.

### 3.2 Genomic sequencing

Table 1 lists candidate variants resulting from WGS analysis that were prioritized by the bioinformatics pipelines for expert review for each of the 34 patients. All variants in Table 1 were confirmed by Sanger sequencing or by clinical genetic testing. (Potentially damaging variants and de novo variants found in cardiac genes are listed in Table S2; those not included in Table 1 were not validated and the relationship with the phenotype in our patients was not known.)

In patients 8 and 24, a de novo variant found in each case was considered to be causal for the condition. In patient 8, WGS performed through the research study revealed a novel heterozygous variant, c.5051‐1G>A, in *CHD7*. Heterozygous mutations in *CHD7* are associated with two defined syndromes, CHARGE syndrome (coloboma, heart defect, atresia choanae, retarded growth and development, genital abnormality, and ear abnormality) (OMIM #214800) and hypogonadotropic hypogonadism 5 with or without anosmia (OMIM #612370). Clinical testing for *CHD7* in this patient, performed after enrollment in the research study, identified the same mutation; this clinical testing was only performed after clinical evaluation revealed the characteristic semicircular canal hypoplasia. The variant alters the last base of intron 22, in the canonical acceptor site, and is predicted to affect splicing. The G nucleotide at that position in the reference sequence is completely conserved across mammalian species. Patient 8 had choanal atresia, hypoplasia of the semicircular canals, and tetralogy of Fallot. As her features were consistent with phenotypes described in the literature for *CHD7*‐associated CHARGE syndrome, we felt that the variant was the causal. Patient 24 has a de novo variant in *JAG1,* c.551G>A (p.Arg184His), found both by WGS and by commercial clinical testing initiated after research participation. This patient was suspected of having tetralogy of Fallot prenatally and after delivery was found to have jejunal atresia and microcolon. Persistent cholestasis and a triangular‐shaped face directed genetic testing toward Alagille syndrome. The variant is considered pathogenic since it has been reported previously in other patients with Alagille syndrome (Krantz et al., 1998) and has been demonstrated to impair protein folding (Tada, Itoh, Ishii‐Watabe, Suzuki, & Kawasaki, 2012). We believed this variant to be causative of this patients’ condition.

An apparent de novo deletion in patient 3 in the gene *NIPBL,* c.5863‐180_5971+111del, was identified by a proprietary pipeline (Personalis, Inc., Menlo Park, CA USA, 10/03/2014). This genomic region showed a decrease in sequencing coverage in the proband as expected for a heterozygous deletion, but there was no support in the mapped reads for a breakpoint, and heterozygous variants within the region suggested that two alleles were present (Fig. S1). This region was previously identified as being difficult to amplify (Gillis et al., 2004). PCR, using the Q5 High‐Fidelity master mix (New England Biolabs Inc. Ipswich, MA, USA), confirmed the absence of a deletion in that region, a result also supported by Sanger sequencing (Fig. S2 and Appendix S1). This suggests that the suggested deletion is a false positive. *NIPBL* mutations are associated with Cornelia de Lange syndrome, with features that include growth retardation, a distinct facial phenotype, hirsutism, upper extremity malformations, cardiac defects, and mental retardation (Landrum et al., 2016). Our patient had neither the typical facial features nor the upper limb anomalies seen frequently in this syndrome, consistent with the experimental result that this variant is likely to be a false positive. The patient was also the infant of a diabetic mother, which is an environmental teratogen associated with fetal cardiac defects (Corrigan, Brazil, & McAuliffe, 2009).

Two patients were found to have an inherited VUS that segregated with the disorder in the family. Patient 11 had a c.722C>T (p.Ser241Phe) variant in *MYH7*. The variant is a novel missense variant located in exon 8 of the *MYH7* gene. The gene encodes beta‐myosin heavy chain which plays a role in sarcomere formation and cardiac muscle function. Variants in *MYH7* have been previously reported in association with a variety of cardiac anomalies including hypertrophic, dilated and restrictive cardiomyopathies, Ebstein anomaly, and left ventricular noncompaction (Abbasi et al., 2016). The patient was diagnosed with Ebstein anomaly; her father, who has mild Ebstein anomaly, was found to carry the variant as well. This variant was not found in ExAC. Using the ACMG InterVar classification tool, this variant was considered of uncertain significance (Li & Wang, 2017). In patient 34, a variant was found in exon 2 of *GATA6*, c.C15G (p.Asp5Glu), which was inherited from his father who has a bicuspid aortic valve. *GATA6* mutations have been associated with pancreatic atresia as well as congenital heart malformations, including atrial septal defect, ventricular septal defect, tetralogy of Fallot, pulmonic stenosis, transposition of the great vessels, tricuspid atresia, and double outlet right ventricle (Digilio & Marino, 2016; de Souza, Mergener, Huber, Campos Pellanda, & Riegel, 2015). This nucleotide (c.C15G) position is conserved in mammals, but the amino acid substitution (p.Asp5Glu) is conservative. The variant is not found in public databases. Using the ACMG InterVar classification tool this variant was therefore considered of uncertain significance.

Eight patients had candidate variants that were also detected in an unaffected parent and are considered variants of uncertain significance because of lack of segregation with phenotype and insufficient literature support for pathogenicity:

1. Patient 1 was found to have the variant c.899‐2A>G in *MYH6*. *MYH6* has been associated with atrial septal defect (Posch et al., 2011; Priest et al., 2016), which is the cardiac defect observed in this patient. The variant was also found in the self‐reported unaffected mother, and is not found in public databases. Using the ACMG InterVar classification tool, this variant is considered of uncertain significance.
2. Patient 2 had a variant in *CITED2*, c.574A>G (p.Ser192Gly). Mutations in *CITED2* have been associated with a variety of congenital cardiac defects (Sperling et al., 2005). This variant, found in exon 2, has a minor allele frequency of 0.001 in ExAC and was previously reported in a patient with aortic stenosis (McDermott, Fong, & Basson, 2015). SIFT (Kumar, Henikoff, & Ng, 2009) and PolyPhen2 (Adzhubei et al., 2010) in silico models predict that the variant would not have a deleterious effect on protein function. This variant was also found in the patients’ self‐reported unaffected mother. Using the ACMG InterVar classification tool this variant is considered likely benign. Due to conflicting reports in the literature (Xu et al., 2014), we consider this a variant of unknown significance.
3. Patient 5 had the variant c.926A>G (p.Asn309Ser) found in *FOXL1*. *FOXL1* is a gene of interest in cardiac defects, particularly hypoplastic left heart, and other congenital anomalies similar to those seen in VACTERL; currently the role of *FOXL1* in cardiac defects remains vague (Shaw‐Smith, 2010). Patient 5 was diagnosed with hypoplastic left heart syndrome and the variant was inherited from his father who does not have a history of a congenital heart defect. The variant is novel and found in exon 1. It is absent from healthy genome datasets and in silico models predict that the variant is tolerated. Using the ACMG InterVar classification tool this variant is considered of uncertain significance.
4. Patient 6 was found to have a c.1037C>T (p.Ala346Val) variant in *GATA4*. *GATA4* mutations have been associated with atrial septal defects, ventricular septal defects, (Reamon‐Buettner & Borlak, 2005), and complex cardiac defects (Duffy, Overmann, Keen, Clegg, & Daston, 2004). Patient 6 was an infant of a diabetic mother. He was born large for gestational age with coarctation of the aorta, a ventricular septal defect, and renal anomalies including a multicystic left kidney and a hypoplastic right kidney. The variant c.1037C>T in exon 6 is of unknown significance, and is present in the self‐reported unaffected mother. The variant has been previously described in association with congenital heart disease but has also been observed in control individuals, with a minor allele frequency of 0.0018 in ExAC. The 1,000 Genomes Project has observed this variant in 0.1% of European individuals. In silico models, SIFT and PolyPhen2, predict that the variant will not affect protein function. The ACMG InterVar classification tool considers this variant to be of uncertain significance. Patient 9 was found to have two candidate variants in heart‐related genes. One variant is c.397C>T (p.Gln133*) in exon 2 of the *NODAL* gene. *NODAL* is involved in left‐right directionality during organ development (Deng et al., 2014; Noel et al., 2013). Mutations in *NODAL* cause heterotaxy and a variety of cardiac defects including dextrocardia, mesocardia, levo‐transposition of great arteries and atrial isomerisms, and others (Mohapatra et al., 2009).
5. Patient 9 was born with double outlet right ventricle and transposition of the great arteries, but no features of visceral heterotaxy. The variant is not found in public databases. This variant was also found in the patient's mother, who is unaffected. The ACMG InterVar classification tool considers this variant to be pathogenic. Due to lack of penetrance in the mother, we consider this variant to be of uncertain significance. The second candidate variant in patient 9 is c.245A>G (p.His82Arg) in exon 2 of *CITED2*. As noted above, *CITED2* has been associated with cardiac defects. This variant was found in the patient's unaffected father. The family is of Hispanic origin and the frequency of this variant is 0.0011 in Hispanics as reported in ExAC. The ACMG InterVar classification tool considers this variant to be of uncertain significance.
6. Patient 16 had a variant found in *GDF1*, c.G485A (p.Gly162Asp), which was inherited from her self‐reported unaffected father. *GDF1* is associated with heart and vasculature development. Variants in this gene have been seen in patients with defects involving the left ventricular outflow tract, conotruncal region, atrial and ventricular septum, and pulmonary vasculature have been noted, as well as left‐right signaling abnormalities. This variant has been seen previously in a patient with tetralogy of Fallot (Karkera et al., 2007; Zhang et al., 2015). This variant was not found in ExAC, and is found at a frequency of 0.0061 in the African subpopulation in the 1000 Genomes Project. The ACMG InterVar classification tool considers this variant to be of uncertain significance.
7. Patient 23 also had a variant found in *MYH6*, c.C4768A (p.Arg1590Ser) which was inherited from his self‐reported unaffected father. As previously stated, *MYH6* has been associated with atrial septal defects, but may also have a role in hypoplastic left heart, ventricular septal defects, and other cardiac malformations (Jia et al., 2015; Touma et al., 2016). This variant was not found in ExAc, and the 1000 Genomes Project observed this variant in 0.1% of Europeans. The ACMG InterVar classification tool considers this variant to be of uncertain significance.
8. Patient 32 had a variant found in exon 2 of *NKX2‐5*, c.C494T (p.Ala165Val), which was inherited from his self‐reported unaffected father. *NKX2‐5* is associated with cardiac development (Granados‐Riveron et al., 2012). Mutations in this gene are believed to be the cause of several different types of cardiac disease including atrial septal defects, dilated cardiomyopathy, tetralogy of Fallot, ventricular septal defects, and AV block (Lalani & Belmont, 2014). The nucleotide is highly conserved across species, and the amino acid substitution is conservative. This variant was not found in ExAC. The ACMG InterVar classification tool considers this variant to be of uncertain significance.

## 4 DISCUSSION

This report describes our experience using genomic sequencing to analyze variations in known cardiac genes to ascertain an underlying genetic etiology for cardiac defects in a cohort of pediatric patients at a large not‐for‐profit healthcare system. In agreement with previous studies (Iascone et al., 2012; Jia et al., 2015), we found pathogenic or likely pathogenic variants in a minority of patients, 6% (2/34). We found 11 variants of unknown significance in 10 patients, 29% (10/34). Overall, 82% (9/11) of the VUS's were inherited from a self‐reported healthy parent, illustrating the difficulty in variant interpretation for this class of disorders, and the requirement for more study before classification can be definitively assigned to the variants discussed above (Digilio & Marino, 2016). In one case (3), we identified a possible deletion, which proved to be a false positive.

Prior studies have reported a higher diagnostic rate using genetic investigations for syndromic congenital heart disorders than for nonsyndromic patients (Digilio & Marino, 2016; Homsy et al., 2015; Sifrim et al., 2016; de Souza et al., 2015). In 25 nonsyndromic patients, we identified no causative variants and found two pathogenic variants in nine syndromic patients. The difference in the fraction of patients with causative findings between these two groups is not statistically significant (*p* = .087 by Fishers Exact test), likely due to the small number of patients.

One possible impediment to clinical implementation of genome‐wide or larger gene panel analysis is the potential for increased numbers of VUS compared to testing a small number of preselected genes. In our study, our predefined gene list included 206 genes known to be associated with embryonal cardiac development. We found 11 candidate VUS's in 34 patients. The average rate of candidate VUS's per patient is not statistically different from that reported by Iascone et al., who found 25 total candidate VUS in 53 patients when studying five genes (*p* = .40 by Fisher's exact test) or by Jia et al. who found five VUS in 12 index patients when studying a panel of 57 genes (excluding one patient in which not all of the variants were validated), (*p* = .74 by Fisher's exact test). Although there are differences between the studies in the disorders and methodology and a small number of patients evaluated, these results suggest that extending variant analyses to a larger set of cardiac genes may not significantly increase the number of VUS per patient.

Previous studies have observed higher yields from genetic testing for conditions with apparently familial transmission, as compared to families with one affected member. In our cohort, in which 32/34 patients (94%) have no reported family history of cardiac defects, we detected pathogenic variants in two of 34 cases (6%). Siedelmann et al. reported that diagnosis was reached in 26.5% of patients (53 of 200 patients) who had familial cardiovascular disease at statistically higher rate than in our study (*p* = .024 by Fisher's Exact test) (Seidelmann et al., 2017). Consistent with this, in their cohort of patients from families with multiple affected family members with CHD, Jia et al. found candidate pathogenic variants for 42% (5/12) of the index patients (excluding the case with the variant that did not validate), also a statistically significant difference (*p* = .028 by Fisher's Exact test). This suggests that diagnostic yield for CHD may be higher if there are other family members affected, and if an autosomal dominant inheritance pattern is suggested.

There are several reasons why causative variants for the currently unexplained patients may not have been identified. (1) The pathogenic variants might have been excluded by the filtering strategy. To focus on variants that are sufficiently interpretable to be clinically reported, we limited the analysis to variants predicted to impact a protein sequence, but noncoding or miRNA variants may be contributory (J. Li et al., 2013; Touma et al., 2016; Xie, Zhou, Chen, & Ni, 2016). We also required candidate variants to be of low frequency (<1%) in reference populations. However, particularly for disorders with incomplete penetrance, the pathogenic variants could be unexpectedly common in control cohorts. (2) We also limited the findings to those genes with sufficient prior knowledge for a CHD association. Extending the search genome‐wide may identify candidate novel CHD genes, which would require further laboratory‐based investigation and larger numbers of affected patients to clarify their role in cardiac development and human disease. (3) There are technical limitations that prevent reliable detection of all genomic variants, with problematic regions including known cardiac genes, and also challenges in copy number and structural variant calling (Goldfeder et al., 2016). These issues could lead to undercalling or overcalling of variants, as illustrated by the deletion in *NIPBL* in patient 3 that failed validation. (4) In our analysis, we searched for variants assuming single‐gene inheritance patterns, but cardiac defects could arise as a result of an oligogenic process (Priest et al., 2016). (5) The disorders in these individuals may result from contributions from epigenetics, nongenomic factors, or environmental influences; maternal diabetes mellitus is associated with cardiac defects in fetuses, and was noted in the pregnancy histories for several families in our cohort (Corrigan et al., 2009). (6) Causative variants might be below the detection limit in blood, as observed previously in patients of somatic mutations (Braunholz et al., 2015; Huang, Xue, Xu, & Yang, 2013).

Despite an increase in understanding of the underlying genes of CHD, assignment of variant pathogenicity remains challenging. In this study, 83% (11/13) of the total candidate variants from all subjects, all in known CHD genes, were considered to be of unknown significance due to insufficient evidence of pathogenicity in the literature, and the majority of those, 80% (9/11), did not segregate with disease. Cardiac defects have been previously associated with variable expressivity and incomplete penetrance, making interpretation of genomic sequencing results more difficult for this class of disorders (Digilio & Marino, 2016; Jia et al., 2015).

We recommended that the self‐reported unaffected parents who carry candidate variants for their child's condition undergo a cardiac examination including an echocardiogram; however, to date none of the parents have done so. Variable intrafamilial expressivity has been previously reported for genes associated with CHD. Recent publications highlight the importance of these types of postbioinformatic diagnostic assessments (Baldridge et al., 2017).

With genetic testing technologies continually improving, and more options available for genetic examination, there are increasing choices for the clinical investigation of congenital heart disorders. One option for clinicians could be focused gene panels, another option highlighted in our patients could be phenotype‐driven WGS. In our cohort, WGS detected what was found on the clinical gene panels, and also offered a wider examination of a larger gene set. This paper illustrates the low yield and the complexity involving the question of penetrance, both of which we think are important for clinicians as they evaluate clinical testing options and potential yields for their patients. Although our cohort is small, the patients and their cardiac defects are reflective of what a clinician would encounter. As both the sequencing methods and our understanding of disease etiology improve, it is expected that WGS and other next‐generation sequencing techniques will be more reliably applied to a wider range of CHDs, as well as other conditions.

## 5 CONCLUSION

In summary, use of WGS identified clearly pathogenic variants for 6% of our pediatric CHD patients. We found variants of unknown significance in 29% of our patients, of which 82% did not segregate with the self‐reported phenotype of the parents. No candidate explanatory variants were found in known heart genes for the remaining 68%. These results suggest that our current knowledge of CHD pathogenesis and ability to interpret genomic sequencing is sufficient for finding a genetic diagnosis in a minority of patients when applied to patients with cardiac defects in the NICU and inpatient wards at a large community hospital. Most likely, the lack of identification of candidate variants in the majority of patients reflects the complex etiology of cardiac disorders, which could include multiple genetic, environmental, and other contributing factors.

## CONFLICT OF INTEREST

The authors declare no conflicts of interest.
