# Supplementary material for: Experience with genomic sequencing in pediatric patients with congenital cardiac defects in a large community hospital
Source: Mol Genet Genomic Med. 2018 Jan 25;6(2):200–12. doi: 10.1002/mgg3.357 (PMC5902396; doi:10.1002/mgg3.357)
Supplement: Supplementary file 1 [file MGG3-6-200-s001.docx]

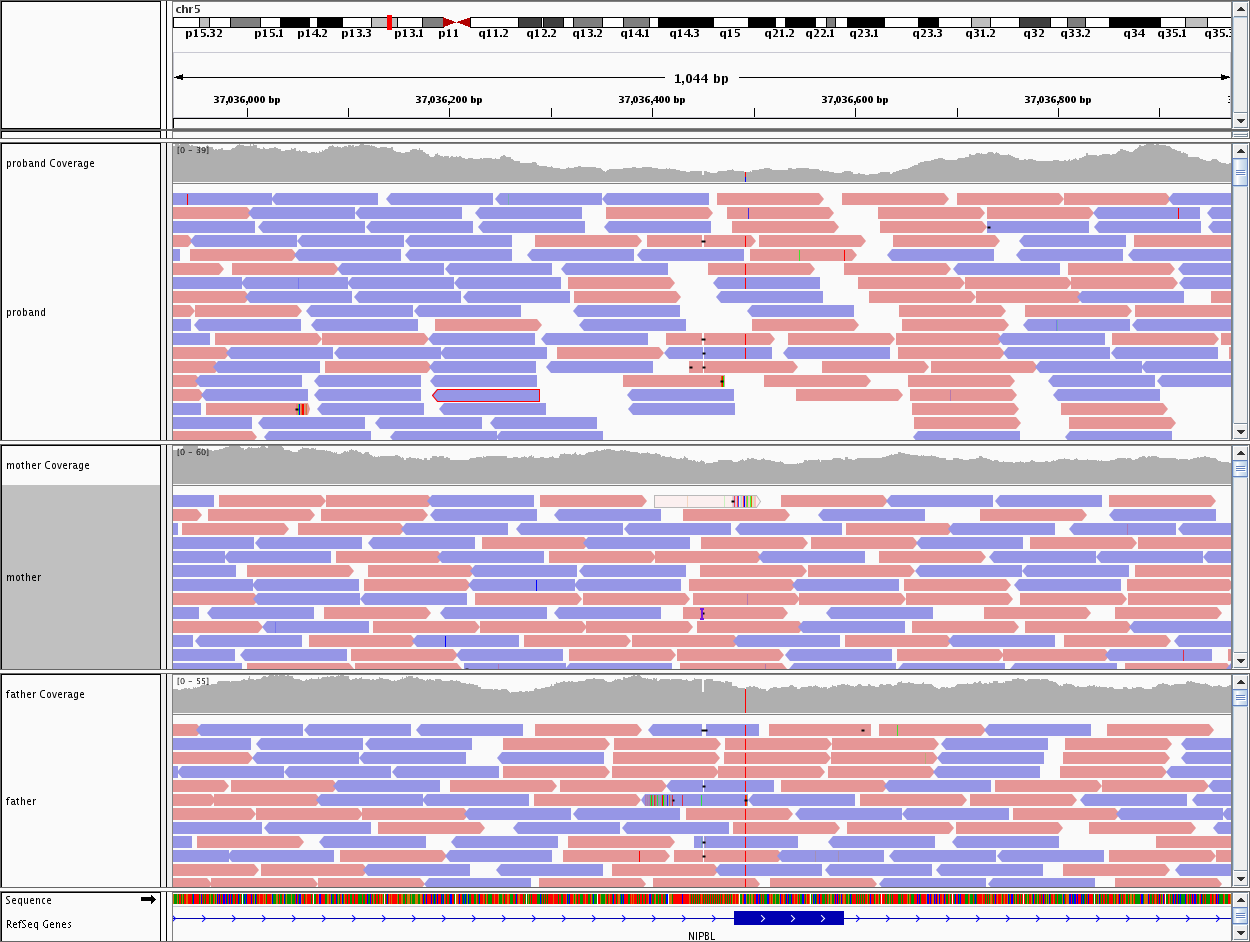


Supplemental Figure 1: Sequencing reads mapped to the genomic region of the potential NIPBL deletion.

The plots show the coverage summary (gray) and the mapped reads (pink and blue) for the proband, mother, and father. Read mappings were obtained from the vendor (Illumina, Inc. San Diego, CA USA). A decrease of coverage is observed in the proband but not the parents, but variants with two alleles are observed in this region in the proband.


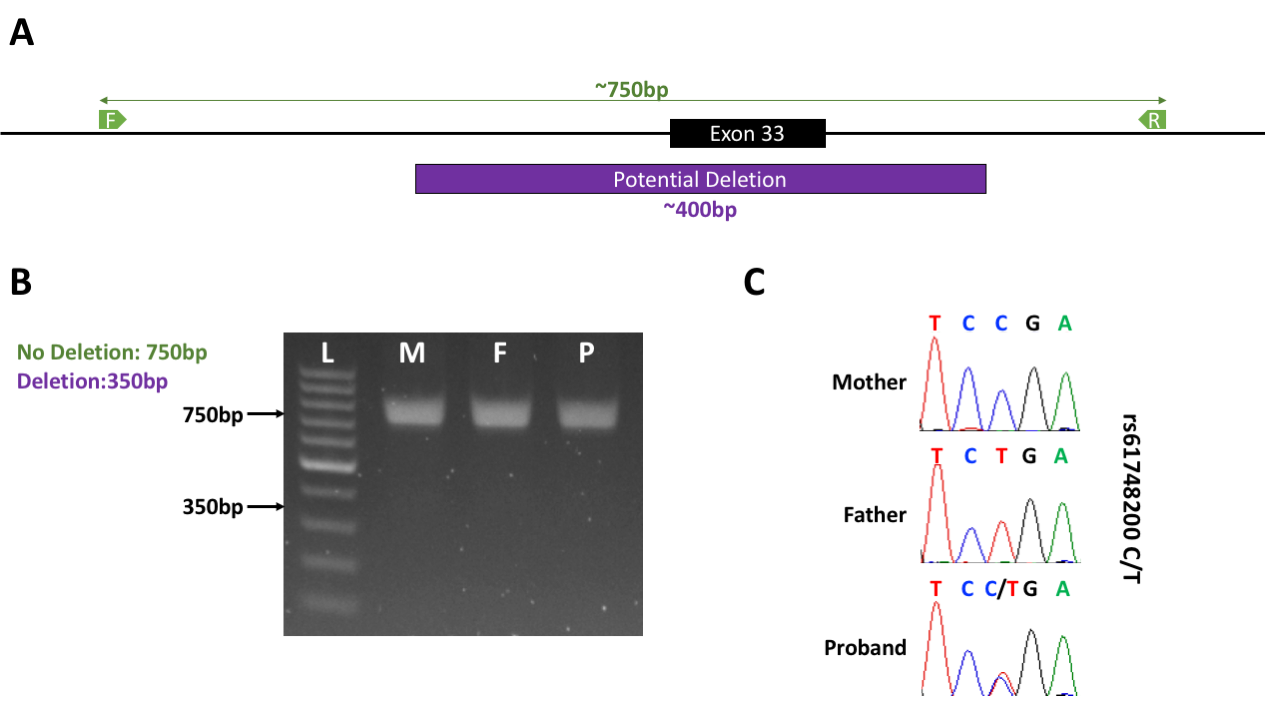


Supplemental Figure 2: Molecular analysis of the potential deletion of exon 33 of NIPBL.

A. Representation of the localization of the potential deletion over exon 33 of NIPBL (NM_015384.4) and relative position of the primers designed to amplify the region. B. Analysis of PCR products in 1.2% agarose gel showing bands at 750bp in all the family members and no band specific of the deletion (350bp) in the proband; L: 100bp ladder, M: mother; F: father; P: proband. C. Chromatogram results of the PCR products showing the rs61748200 (NM_015384.4:c.5874C>T; p.Ser1958Ser) genotypes in the family. The heterozygote status of the proband is confirming the absence of a deletion over exon 33 of NIPBL.

Supplemental Methods: Primers were designed to cover the potential deletion in NIPBL (primer sequences available on request). The PCR was performed using the Q5 High-Fidelity 2X Master Mix (New England Biolabs). After Exo/SAP purification (Applied Biosystems), the amplicons were sequenced by using the BigDye V.3.1 Terminator chemistry (Applied Biosystems) and separated on an ABI 3730xl genetic analyzer (Applied Biosystems). Data were evaluated using Sequencher V.5.0 software (Gene Codes).
